# Clinical and Developmental Outcomes After 50 Years of Newborn Bloodspot Screening for Classical Galactosaemia in the Republic of Ireland

**DOI:** 10.1002/jmd2.70022

**Published:** 2025-05-26

**Authors:** D. Pereira, E. Loftus, C. E. Thompson, F. Boyle, J. McNulty, R. Boruah, E. Crushell, C. Howard, J. Hughes, A. A. Monavari, E. P. Treacy, A. Beegan, N. Jordan, Y. Rogers, A. Collins, J. J. Brady, M. Elsammak, P. D. Mayne, I. Knerr

**Affiliations:** ^1^ National Centre for Inherited Metabolic Disorders, Children's Health Ireland Dublin Ireland; ^2^ Department of Neurodisability Children's Health Ireland Dublin Ireland; ^3^ School of Medicine, University College Dublin Dublin Ireland; ^4^ School of Medicine Trinity College Dublin Dublin Ireland; ^5^ Clinical Research Centre, Children's Health Ireland Dublin Ireland; ^6^ Data Science Centre, School of Population Health, Royal College of Surgeons in Ireland Dublin Ireland; ^7^ Public Health Medicine Health Service Executive (HSE), HSE Area Offices Tullamore Ireland; ^8^ National Newborn Screening Laboratory, Children's Health Ireland Dublin Ireland

**Keywords:** carbohydrate metabolism, cataract, classical galactosaemia, galactose, neurocognitive outcomes, neuro‐development, newborn screening, outcomes

## Abstract

Classical Galactosaemia (CG) is an inborn error of carbohydrate metabolism. In untreated neonates, CG leads to a multi‐organ toxicity with life‐threatening symptoms. Newborn Screening for CG began in the Republic of Ireland in 1972. In Ireland, two forms of neonatal screening occur. High‐risk infants are fed lactose‐free/galactose‐free formula until the result of their Beutler screening test on day 1. All other infants are fed as per parental preference and are screened on day three to five. While immediate or early implementation of a strict lactose‐free diet together with medical interventions will usually address the acute medical complications, long‐term complications are common. We reviewed retrospectively and anonymised the clinical outcomes of our CG cohort, derived from our hospital‐based database. Patient demographic information, co‐morbidities, developmental assessment results, and other relevant health indicators were analysed from birth to 18 years. Out of 217 patients, 95% of subjects were alive at 18 years of age. Common co‐morbidities were speech and language difficulty (43.5%) and learning difficulty (25.5%). In this Irish cohort, Friedreich Ataxia is a genetically linked condition for a subgroup of CG individuals (7.9%). Our data demonstrate that while early diagnosis prevents mortality, it does not prevent developmental disorders, underpinning the neuro‐developmental nature of CG. High‐risk and routine newborn screening for CG have reduced the mortality rate of the disorder, and early medical and dietetic intervention is a success story. However, long‐term medical and developmental challenges persist, and an early, proactive multidisciplinary approach may further mitigate the phenotype in CG patients diagnosed on NBS.

**Abbreviations**
CG: classical galactosaemia
CHI: Children's Health Ireland
D2: Duarte variant
DEXA: dual energy x‐ray absorptiometry
FA: Friedreich's Ataxia
FSIQ: Full‐Scale Intelligence Quotient
GALE: UDP‐galactose 4‐epimerase
GALK: galactokinase
GALM: galactose mutarotase
GALT: galactose‐1‐phosphate uridylyltransferase
HRS: high risk screening
ITC: Irish Travelling Community
LD: learning difficulty
NBS: newborn screening
NBSP: newborn screening programme
NCIMD: National Centre for Inherited Metabolic Disorders
POI: primary ovarian insufficiency
QC: quality control
SLD: speech and language disorder or delay
UMP: uridine monophosphate
WISC‐V: Wechsler Intelligence Scale For Children 5th edition

**Summary**

- Developmental issues may occur in almost half of all children with CG at some stage of their lives.
- Early and regular developmental assessments and early, proactive intervention are recommended to further improve developmental outcomes.
- Further studies are needed to identify further pathophysiology‐oriented treatments.

## 1 Background

Classical galactosaemia (CG, OMIM # 230400) is an inborn error of carbohydrate metabolism caused by severe deficiency in the galactose‐1‐phosphate uridylyltransferase (GALT) enzyme due to pathogenic *GALT* gene variants. The Leloir pathway is the predominant enzymatic pathway in galactose metabolism. In the first step, beta‐D‐galactose is epimerised by the enzyme galactose mutarotase (GALM) to alpha‐D‐galactose. Then, alpha‐D‐galactose is phosphorylated by galactokinase (GALK) to yield galactose 1‐phosphate. The next enzyme of the Leloir pathway is GALT which catalyses the conversion of galactose‐1‐phosphate and UDP‐glucose to glucose‐1‐phosphate and UDP‐galactose. The last step involves UDP‐galactose 4‐epimerase and generates UDP‐glucose [1, 2]. In CG, the near absence of GALT leads to elevated metabolites like galactose‐1‐phosphate, galactitol, and galactonate, which may be toxic [3]. Other findings include abnormal glycosylation and dysregulation of cellular pathways, though the full pathogenesis is not understood [4, 5].

Newborn screening programmes (NBSPs) are crucial to prevent morbidity and mortality which may be associated with inherited metabolic disorders. Intoxication disorders like CG are ideal for NBSPs due to brief symptom‐free periods, specific markers, and better outcomes with early treatment [6]. However, NBSPs face challenges, with varying screening approaches by country [7, 8, 9]. The availability of a diagnostic test distinguishing disease from non‐classical or asymptomatic phenotypes is essential but not always clear [10].

Primary CG NBSP tests include quantitative galactose determination and erythrocyte GALT enzyme activity using a fluorometric assay with high sensitivity and specificity [9]. Many centres use fluorometric Beutler‐based methods or galactose level testing alone or combined with GALT [11, 12]. Combination GALT enzymology and DNA testing in some regions reduce false positives [13]. Elevated erythrocyte galactose‐1‐phosphate and genetic testing for *GALT* variants are also used [13]. Comparing methodologies is challenging due to NBSP standardisation, with no direct comparison studies [14, 15]. Reference range changes impact specificity [16], but CG NBSP sensitivity remains high in previous studies [13, 16].

In Ireland, the CG NBSP was introduced in 1972 using total galactose testing through bacterial inhibition assay, later replaced by a colourimetric enzymatic assay in 2011–2012. A second‐tier confirmatory test for GALT enzyme activity using the Beutler method was added in 1976 [10]. High‐risk screening (HRS) began in 1996 [17], with high‐risk infants screened on the first day of life with 1st tier GALT activity analysis by Beutler and fed non‐lactose formula until results become available. HRS is offered to all babies born to Traveller parents and to siblings of known cases. For other infants, standard NBS occurs on days 3–5, measuring total galactose with second‐tier GALT enzyme activity if levels are high. High‐risk infants include those from the Irish Traveller Community (ITC) and those with CG family history. Cut‐off thresholds for galactose are amended quarterly, if warranted, based on the 97th percentile. Two levels of commercial internal quality control (QC) material are run with each batch of total galactose samples. For the Beutler assay, previous samples known to have “no activity present” or “activity present” are retested for quality assurance purposes. The laboratory participates in an external quality assessment scheme for the CG assays and follows multirule QC procedures.

Reporting accurate Irish NBS enrolment rates is challenging due to a lack of end‐to‐end automation but are estimated above 99%. Annually, about 1680 HRS and 60,800 NBS are performed. Some HRSs are for reasons other than ITC ethnicity or CG family history, such as impending blood transfusions.

Classical Galactosaemia typically presents in infancy as a potentially life‐threatening disease. Affected infants consuming breast milk or lactose‐containing formula usually develop life‐threatening multi‐organ complications within days. Signs and symptoms include poor feeding, faltering growth, hepatomegaly, conjugated hyperbilirubinemia, umbilical stump bleeding, hypotonia, and hypoglycaemia [18]. Coagulopathy, transaminitis, and septicaemia are cardinal features. Prompt treatment, involving supportive care and immediate removal of lactose‐based foods, typically reverses all acute symptoms and significantly reduces complications and death [18, 19].

Following diagnosis confirmation, a life‐long lactose‐free andgalactose‐restricted diet (in young infants also galactose‐free) is recommended for management of CG. Those affected are recommended to exclude lactose from milk and milk products of mammalian origin. In Ireland, galactose from non‐milk sources, for example, fruits, vegetables, legumes, or offal, is not restricted. Studies show that patients with CG following a less strict diet may develop fewer neurological complications [20]. A previous report from our centre reported improvements in the glycosylation patterns in a subset of children with CG after short‐term galactose supplementation [21]. Nutritional assessments focus on calcium, iodine, and vitamin D, as decreased bone health is common in CG [22, 23, 24, 25]. This is likely multifaceted, possibly linked to abnormal collagen O‐glycosylation and other alterations of bone metabolism, adherence to a lactose‐free diet without adequate micronutrient supplementation, and primary ovarian insufficiency (POI) in females [26].

Despite early treatment, individuals with CG often develop long‐term medical and developmental complications [27]. Most females with CG exhibit POI, with up to 80% prevalence in older populations [20]. Gonadal function in males appears unaffected [28], likely due to higher levels of galactose metabolising enzymes in ovaries compared to testes [29, 30]. POI consequences include amenorrhea, oligomenorrhea, delayed or absent pubertal development, subfertility, and infertility [31], though successful pregnancies can occur [32].

Adverse developmental outcomes are common. Studies consistently show most individuals with CG exhibit neurocognitive impairments and generally function within a lower IQ range than healthy comparisons [20, 33]. Speech motor issues, childhood apraxia of speech, dysarthria, and impairments in grammar and vocabulary are prevalent, with 35%–66.5% affected ([20, 27, 34, 35, 36]). Additionally, motor disorders, particularly strength and coordination issues and tremors, are common in children and adolescents with CG [20, 37].

More than 300 variants of the *GALT* gene are linked to CG and GALT deficiency, with the two most common pathogenic variants, c.563A>G, p.Q188R and c855G>T, p.K285N accounting for over 70% of CG‐causing variants in Caucasian populations internationally [20, 38]. Studies show that the c.563A>G (p.Q188R) genotype may account for 92%–94% of galactosaemic alleles [17]. In the ITC, an indigenous ethnic minority group in Ireland, this allele accounts for 100% of CG cases, with an incidence of about 1:340 [39]. This variant is generally associated with a severe phenotype, though smaller cohort studies show conflicting evidence [40, 41]. In Black Americans with CG, S135L is a common variant rarely seen in Caucasians [42]. While long‐term outcomes for individuals homozygous for the S135L variant of *GALT* may be milder, complications can still occur [43, 44].

## 2 Methods

This single‐centre, retrospective, longitudinal study analysed biochemical, genetic, clinical, and developmental data from a national cohort of patients aged 0–18 years with CG over a 50‐year period. Conducted at the National Centre for Inherited Metabolic Disorders (NCIMD) in Dublin, Ireland, the study ran from August 2020 to June 2022. Our centre is based at Children's Health Ireland at Temple Street in Dublin where care is provided for all paediatric galactosaemia patients in the Republic of Ireland.

Institutional ethical approval was provided for this research study by the CHI Research Ethics Committee.

### 2.1 Subjects

Patients with CG diagnosed through NBSP from 1972 to 2022 were identified via the Galactosaemia Register and national NBSP data, both based at NCIMD. Those diagnosed through other means or abroad and later managed at NCIMD were identified via the Galactosaemia Register. Initially, 245 patients (ages 0–18 years.) were included. Data was collected from initial contact until age 18, discharge, or most recent available data. After reviewing medical records, 28 patients with non‐classical diagnoses, including Duarte Galactosaemia or heterozygous carriers, were excluded; these false positive screening results came from both NBS (*n* = 20) and HRS (*n* = 7). Thus, 217 patients with CG were included, representing the total number of paediatric patients diagnosed with or managed for CG in the Republic of Ireland from 1972 to June 2022.

### 2.2 Data Points Collected

Data points were selected based on the clinical and biochemical parameters of this study. A list of all 24 data points is available in Table 1. This data was collected from paper‐based or electronic medical records.

**TABLE 1:** Data completeness for each data point collected.

Data point	Data completeness (*n*, %)
Gender	100% (*n* = 217)
Ethnicity	100% (*n* = 217)
Family history of CG	100% (*n* = 217)
Sibling with CG	100% (*n* = 217)
Date of Birth	99.5% (*n* = 216)
Age at clinical presentation	92.2% (*n* = 200)
Diet prior to diagnosis	96.3% (*n* = 209)
Age at diagnosis	94.5% (*n* = 205)
Genotype	85.7% (*n* = 186)
Outcome at 18 years old	85.7% (*n* = 186)
Coagulopathy at presentation	81.1% (*n* = 176)
Hepatic dysfunction at presentation	81.1% (*n* = 176)
History of fracture	81.1% (*n* = 176)
Bone density assessments	81.1% (*n* = 176)
Cataracts present	80.6% (*n* = 175)
Primary ovarian insufficiency	80.2% (*n* = 174)
Learning difficulty at > 5 years old	79.7% (*n* = 173)
Motor issues	78.8% (*n* = 171)
Sepsis at clinical presentation	78.3% (*n* = 170)
Type of sepsis	78.3% (*n* = 170)
Speech and language difficulty at > 5 years old	77.9% (*n* = 169)
Age at first ophthalmology review	71.0% (*n* = 154)
Medical comorbidities	69.6% (*n* = 151)
Age at genetic testing	59.9% (*n* = 130)

### 2.3 Statistical Analysis

Data was collected and patient characteristics summarised using a Microsoft Excel spreadsheet and descriptive statistics were applied. Missing data was documented and accounted for in statistical analysis, and single imputation procedures were applied where appropriate. Data quality for categorical variables was reviewed, and one‐hot‐encoding and data cleansing were used with missing values set as NaN (“not a number”); Boolean encoding was used where appropriate and Python libraries were used to structure datasets. Fisher's exact test, or Chi square test, and the two‐proportion test were used as appropriate. Linear correlation between two variables was measured using Pearson correlation coefficient. Odds ratios (OR) and confidence intervals (CI) were calculated, and Firth‐type logistic regression was applied as appropriate. Multivariate analysis of variance (MANOVA) was used to test the effect of multiple independent variables on outcome. For statistical procedures IBM SPSS Statistics software and Jupyter Notebook were used. Significance levels were set at *p* ≤ 0.05.

### 2.4 Aim

The aims of this study were to report on:

1. The natural history of CG in a national cohort of patients aged 0–18 years
2. Survival to adulthood in children with CG (primary outcome)
3. Survival to adulthood without developmental difficulties in patients with CG (secondary outcome)
4. The relationship between survival to adulthood with/without developmental disability and a number of clinical variables: age at diagnosis, clinical condition at diagnosis, and mode of diagnosis.

## 3 Results

### 3.1 Demographics

**FIGURE 1:**
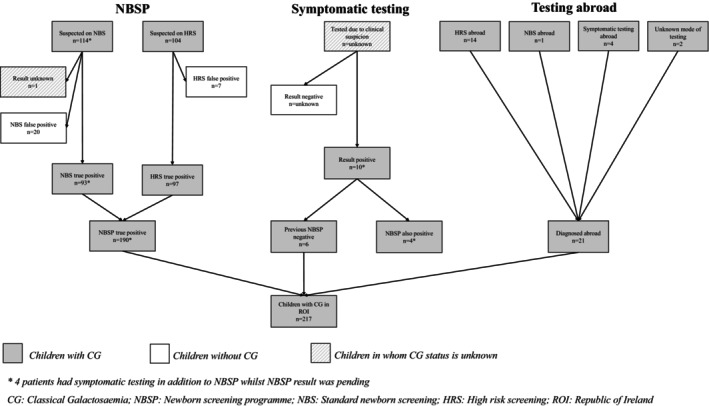
Diagnostic pathway of the Classical Galactosaemia cohort in the Republic of Ireland. *Four patients had symptomatic testing in addition to NBSP while NBSP result was pending. CG: classical galactosaemia; HRS: high‐risk screening; NBS: standard newborn screening; NBSP: Newborn screening program; ROI: Republic of Ireland. 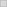 Children with CG, 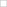 Children without CG, 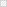 Children in whom CG status is unknown.

Between 1972 and April 2022, CG was suspected on NBSP in 224 patients (53.1% male, *n* = 119) aged 0–18 years and confirmed in 190. Six additional patients were diagnosed symptomatically after a false‐negative NBSP result, giving a birth prevalence of 1 in 17 936 [45]. Twenty‐one more patients were diagnosed abroad (i.e., Irish General and ITC), totaling 217 CG patients nationally, with a point prevalence of 4.04 cases per 100 000 [45]. Figure 1 shows the diagnostic pathway for this cohort.

Classical Galactosaemia is inherited in an autosomal recessive pattern. Of those who attended for a clinical consultation (*n* = 217), a family history of CG was noted at the initial consultation in 54.8% (*n* = 119) of cases; 40.3% of these involved an older sibling (*n* = 48), with the remainder relating to second degree relatives (59.7%; *n* = 71). Patients born in Ireland with a family history were eligible for formal HRS from 1996, with an 88% enrolment rate (*n* = 81) and higher rates among ITC individuals compared to others with 1st grade relatives. See Table 2 for details.

**TABLE 2:** Enrolment rates to the high‐risk screening program in the Republic of Ireland.

Population, born in Ireland	% (*n*) enrolled to HRS 1972–2022	% (*n*) enrolled to HRS 1972–1996	% (*n*) enrolled to HRS 1996–2022
ITC ethnicity	73% (84/115)	27.3% (9/33)	91.5% (75/82)
Family history of CG	75.5% (83/110)	44.1% (15/34)	89.5% (68/76)
Older sibling with CG	77.8% (35/45)	56.5% (13/23)	100% (22)

Abbreviations: CG: classical galactosaemia; HRS: high‐risk screening; ITC: Irish Traveller community.

For the 217 CG patients, age at diagnosis was known for 94.5% (*n* = 205). Infants who underwent HRS had better clinical status at presentation and significantly lower odds of death compared to those identified through NBS (OR: 0.127, 95% CI: 0.027–0.598, *p* < 0.009). See Table 3 for age at diagnosis by cohort.

**TABLE 3:** Age at diagnosis in the paediatric classical galactosaemia population.

Cohort	Age at diagnosis, days	Range (days)
	Mean (median)	
Total cohort with known age at diagnosis *n* = 205	8 (4)	1–544
NBS ROI *n* = 87	7 (6)	2–19
HRS Ireland *n* = 96	2 (1)	1–8
Abroad *n* = 12	10 (9)	1–28
Symptomatic testing in ROI (*n* = 10)	76 (19)	2–544

Abbreviations: CG: classical galactosaemia; HRS: high‐risk screening; NBS: newborn screening; ROI: Republic of Ireland.

Most patients, 61.3% (*n* = 133) were from the ITC, 37.8% from the general Irish population (*n* = 82), and 0.01% from other ethnic backgrounds (*n* = 2). The ITC, a nomadic Caucasian group from the British Isles with distinct cultural traditions, constitutes 0.66% of the Irish population but 61.3% of the CG population [46, 47]. Their rights are protected in Irish policy [48], and high consanguinity rates, along with increased prevalence of inherited metabolic disorders and socioeconomic disadvantages, are noted [49, 50, 51].

Since the HRS programme began in 1996, 91.5% (*n* = 75) of ITC patients with CG in Ireland underwent screening. Additionally, 77.8% (*n* = 14) of ITC patients diagnosed in the UK also underwent HRS.

### 3.2 Outcomes

Clinical outcomes were available for 71.9% of the cohort (*n* = 156), including those still attending the service (43.3%, *n* = 94), transitioned to adult services (24%, *n* = 52), and those who died before 18 years of age (4.6%, *n* = 10). The remainder were lost to follow‐up (11.1%, *n* = 24), had missing or incomplete records (14.3%, *n* = 31), or moved abroad (2.8%, *n* = 6). Most of these were from the ITC (83.3%, 74.1%, and 100%, respectively).

4.6% (*n* = 10) of the cohort died in childhood. All deaths occurred in the neonatal period. Six were from the ITC. Five infants died before the NBS card was received in the first 10 years of the NBSP, with clinical details about the deaths unknown. Among the remaining five, four were symptomatic at diagnosis and died of galactosaemia‐related issues, while one, diagnosed via HRS, died in the neonatal period, though details are unavailable. The median age at diagnosis was 7 days (range 1–16 days). Genotype data was available for four patients, all homozygous for the common Q188R variant; another likely had this variant due to known family history. Five patients from the first decade of NBSP lacked genotype data. Female sex was associated with reduced odds of death compared to males, but this was not significant (OR: 0.622, 95% CI: 0.156–2.484). HRS was associated with better clinical outcomes and reducing odds of death compared to NBS (OR: 0.127, 95% CI: 0.027–0.598, *p* < 0.009).

**FIGURE 2:**
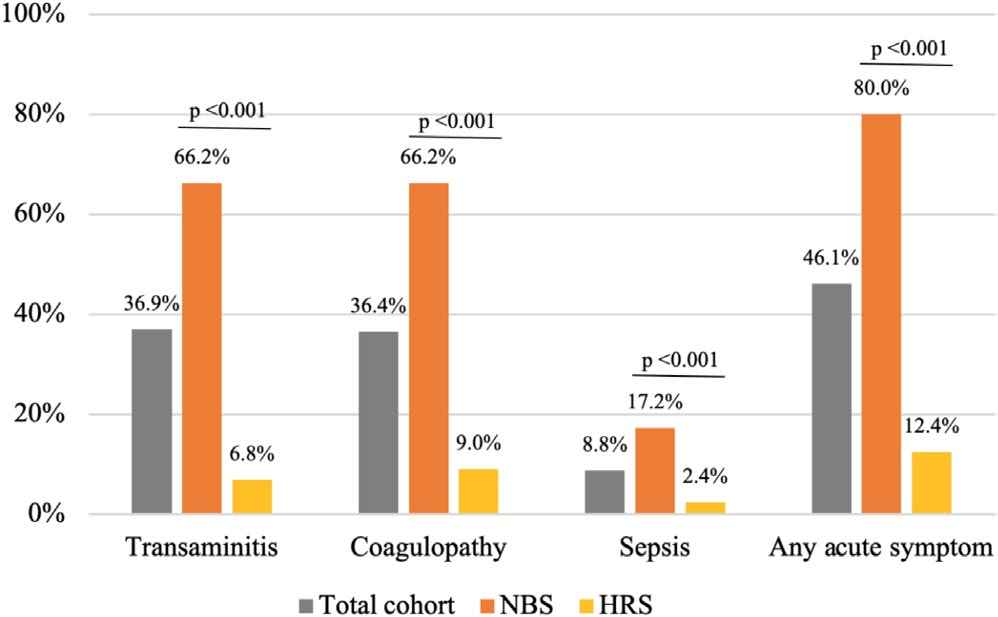
Presence of acute intoxication symptoms at diagnosis in infants with classical galactosaemia. CG: classical galactosaemia; HRS: high‐risk screening; NBS: newborn screening.

Clinical data on transaminitis, coagulopathy, or sepsis at presentation was available for 78.8% (*n* = 171). Results of acute symptoms and subgroup analysis is shown in Figure 2, with the two‐proportion test demonstrating higher rates of statistical significance of all acute intoxication symptoms in the NBS compared to the HRS group. Transaminitis was defined by ALT, AST, or conjugated bilirubin greater than or equal to three times above the reference range threshold for the testing site at that time, or a diagnosis of transaminitis or liver dysfunction made by the referring or treating clinician. This was present in 29.9% (*n* = 65). Coagulopathy was defined by APTT, PT, or INR above the reference range threshold for that testing site, presence of a bleeding diathesis, or a clinical diagnosis of coagulopathy documented by the clinician. This was present in 29.5% (*n* = 64). Sepsis was indicated by positive blood cultures or high clinical suspicion resulting in IV antibiotics for 5 days or more. Sepsis was present in 6.9% (*n* = 15) and types included *E. coli* (*n* = 11), gram‐positive (*n* = 2), other gram‐negative (*n* = 1), and clinically suspected (*n* = 1). Infants identified through HRS had a lower percentage (8.98%) of coagulopathy at diagnosis than those identified by NBS (66.15%); this was significant (*p* < 0.001; 95% confidence interval difference [0.44, 0.7]). Infants who received soy‐based feeds only had a lower percentage (8.51%) of coagulopathy at diagnosis than those with other diets (71.42%); this was significant (*p* < 0.001; 95% confidence interval difference [0.51, 0.74]).

#### 3.2.1 False Negative NBS

Six patients had false‐negative NBS for CG but were later diagnosed based on clinical suspicion and testing. We are not aware of patients with false‐negative results who have died. Clinical presentation data was available for 83.3% (*n* = 5); all showed biochemical transaminitis, 20% had coagulopathy, and 20% had sepsis. All cases occurred before 2010 when the colourimetric enzymatic assay replaced the bacterial inhibition assay for total galactose quantification. Possible contributing factors include, for example, poor feeding prior to NBS in two cases and non‐lactose‐containing feed in one case. GALT activity through Beutler testing was < 0.5gsubs/h per gram of haemoglobin in all cases, and confirmatory genetic testing demonstrated Q188R/Q188R in two cases, S135L/S135L in one case, and Q188R/F194L, Q188R/R333W, and Q188R/K127E in the remaining three cases.

#### 3.2.2 Genotype

Most patients had the common *GALT* Q188R variant in a homozygous state (80.2%, *n* = 174). Other variants included one case each of: Q188R/F194L, Q188R/R333W, Q188R/Y89D, Q188R/K127E, Q188R/S143L, Q188R/Q200fs, S143L/S143L, and compound heterozygosity (c.982C>T/c.329‐2A>C). Individuals who did not meet criteria for having CG (*n* = 28) were children with *GALT* Duarte‐2 variants, including N314D/Q188R and N314D/N314D.

Genetic testing was incomplete for 6% (*n* = 13), with six having a first‐degree relative with Q188R/Q188R. Genotype data was missing for an additional 8.3% (*n* = 18). There was no significant relationship between *GALT* Q188R and odds of developmental disorders compared to non‐homozygous individuals. In CG patients with neurological or cardiac symptoms, analysis of frataxin gene and whole exome sequencing was used when available.

#### 3.2.3 Developmental Assessment, Screening, and Clinical Outcomes

Paediatric patients are seen at NCIMD, predominantly in a specialised multidisciplinary Galactosaemia clinic, for follow‐up until 18 years old. Early developmental progress was assessed through history and clinical exams, with formal psychological evaluations conducted if developmental concerns arose. Over 50 years, many patients received non‐standardised developmental screening at ages two (*n* = 136), three (*n* = 129), and five (*n* = 119). Formal standardised developmental or cognitive assessments were conducted for 40.1% (*n* = 87) of patients during their childhood. Psychometric tools used in this process evolved over time, varying based on the era and needs of the child; these included the Wechsler Intelligence Scale For Children (WISC), Wechsler Preschool and Primary Scale of Intelligence, and Stanford Binet assessment.

Learning difficulty (LD) and speech/language difficulties (SLD) were found in 26.6% (*n* = 47) and 43.5% (*n* = 77) of patients aged five and older who underwent clinical follow‐up (*n* = 177), respectively. LD was defined by an FSIQ below 70, special needs schooling, or any specific learning impairments. SLD was defined by a diagnosis of speech disorder using tests such as the Clinical Evaluation of Language Fundamentals Preschool‐3, Preschool Language Scales, or Renfrew Action Picture Test, or significant deficits at age five or older using non‐standardised clinician evaluation.

Other developmental issues included unexplained motor disorders (4%, *n* = 7), such as ataxia, tremor, or developmental coordination disorder. Among those with unexplained ataxia, two siblings who also had a learning difficulty (intellectual disability diagnosed through psychological assessment) had similar neuroimaging findings, including cerebellar atrophy, white matter T2 hyperintensities, and hypomyelination. All had negative genetic testing for FA.

**FIGURE 3:**
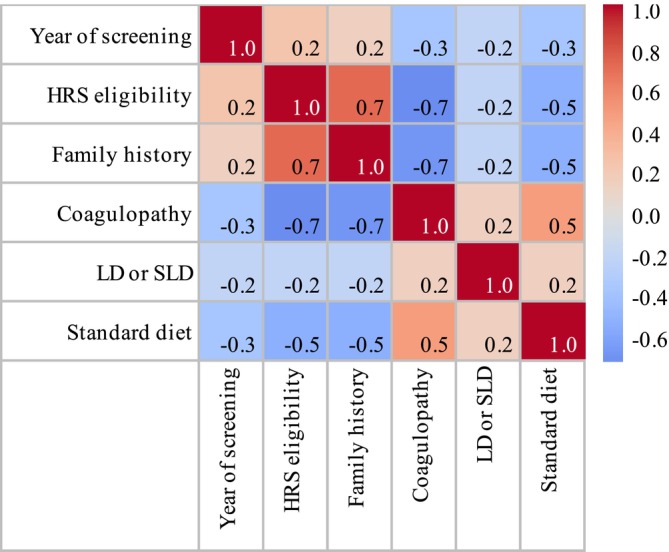
Correlation heatmap showing Pearson correlation coefficients between numerical variables in the paediatric classical galactosaemia population. Standard diet: Breastmilk or cows‐milk infant formula pre‐diagnosis. CG: classical galactosaemia; HRS: high‐risk screening; LD: learning difficulty; NBS: newborn screening; SLD: speech and language difficulty.

There was significant overlap between LD, SLD, and motor disorders, with 47.8% (*n* = 85) having at least one of these difficulties. Significant associations were found between the year of birth and survival without developmental issues (*p* = 0.0233), that is pointing towards more comorbidities in earlier years of the screening program. Other factors associated with an increased incidence of developmental issues were having no family history of CG (*p* = 0.029) which is likely directly due to the impact of HRS eligibility. There was no significant difference in developmental outcome between genders. Having any coagulopathy was associated with a significantly increased odds of developmental issues in childhood (OR: 3.3, 95% CI 1.536–7.091, *p* = 0.014). There was a trend towards lower rates of LD, SLD, and motor issues in the ITC population compared to the non‐ITC population (40.9% and 57.5% respectively); the statistical significance of the relationship between ethnicity and developmental outcome could not be confirmed given the strong overlap between screening modality and ethnicity. A graphical representation of Pearson correlation coefficients between the different variables is shown in Figure 3.

**FIGURE 4:**
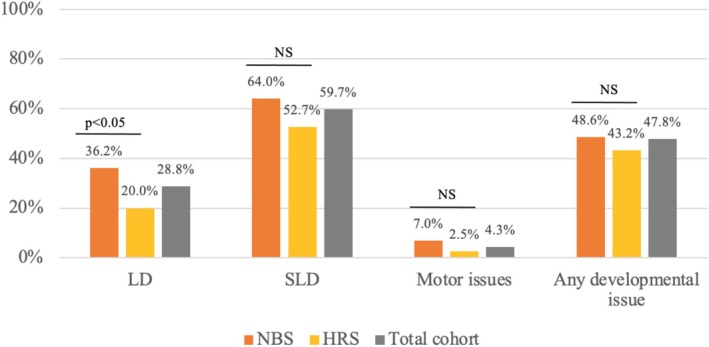
Neurocognitive outcomes in children over 5 years old with classical galactosaemia. CG: classical galactosaemia; HRS: high risk screening; LD: learning difficulty; NBS: newborn screening; NS: No significance; SLD: speech and language difficulty.

Patients diagnosed pre‐symptomatically and on HRS had a tendency towards lower rates of developmental issues. The two proportion test demonstrated statistically significant lower rates of LD in the HRS group compared to the NBS group (*p* = 0.034) as shown in Figure 4.

#### 3.2.4 Medical Co‐Morbidities

Clinical screening for CG complications has evolved over 50 years, influenced by improved knowledge, funding, and healthcare changes. Complete clinical information about medical co‐morbidities was available for 53% (*n* = 115) patients, partial clinical information for a further 28.6% (*n* = 62) patients, and no clinical information for 18.4% (*n* = 40) patients.

##### 3.2.4.1 Cataracts

**FIGURE 5:**
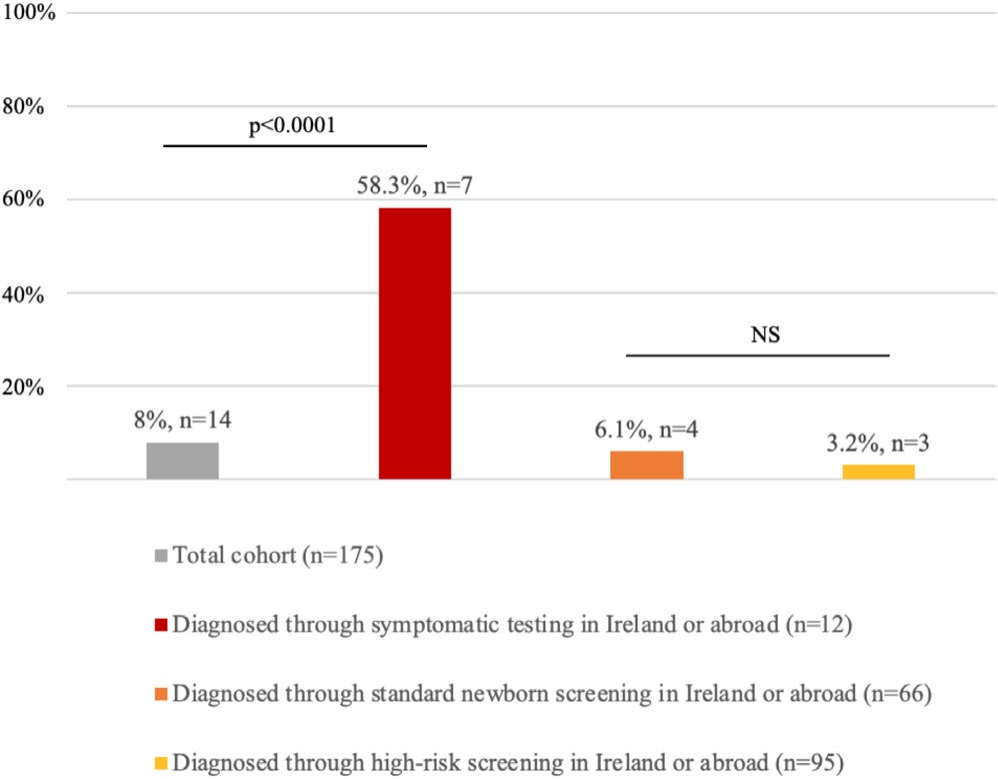
Cataract prevalence throughout childhood in the paediatric classical galactosaemia population. *Note:* The diagnostic pathway was unknown for 2 children, and that only children for whom ophthalmic data was available are represented in this chart. NS: No significance.

Among patients with cataract data available (*n* = 175), childhood prevalence was 8% (*n* = 14). Cataracts were associated with Q188R/Q188R status using Fisher's exact test (*p* < 0.004). There was a trend towards older age at diagnosis (range 1–544 days, median 9 days) in those with cataracts. Chi2 analysis and post hoc pairwise comparisons demonstrated a statistically higher cataract prevalence in children diagnosed with CG through a symptomatic testing pathway (58.3%) than those diagnosed through NBS (6.1%) or HRS (3.2%) (*p* < 0.0001). No significant difference was found between NBS and HRS cataract prevalence (*p* = 0.62). See Figure 5 for more results.

##### 3.2.4.2 Osteopenia

Bone health screening was limited in the early years due to awareness and resources. 18.9% (*n* = 41) had a dual energy x‐ray absorptiometry (DEXA) scan in childhood, 10.6% (*n* = 23) had childhood osteopenia, and 3.2% (*n* = 7) had one or more fractures and a subsequent bone health assessment.

##### 3.2.4.3 Friedreich's Ataxia (FA)

A subset of patients with CG and comorbid FA, a unique genotype and phenotype within the ITC, was identified. Patients diagnosed with FA had confirmatory genetic testing for FA when clinical suspicion arose or in case of a sibling with CG and FA. The prevalence was 6.7 per 100 patients with CG, all from the ITC. Regular neurological examination is recommended for CG patients due to FA prevalence and neurological risks [52, 53].

##### 3.2.4.4 Primary Ovarian Insufficiency

Data on ovarian insufficiency and fertility is limited due to changes in data collection practises and screening methods, and due to the 0–18 year age range of the cohort. Of the 106 female patients in the cohort, 59 were aged 16 years or older during the study period, of which 20.3% (*n* = 12) were diagnosed with POI. This figure almost certainly under‐reports the incidence of POI in the paediatric CG cohort.

## 4 Discussion

This 217‐patient CG cohort is the largest single‐centre cohort described to date, providing valuable insight into the longitudinal outcomes of children with CG. Over half of the cohort were ITC members, differentiating it from other studies and necessitating consideration when reviewing outcomes. Given the significant ITC population abroad, including over 54 000 in the UK, these findings may be relevant to families, clinicians, and researchers outside Ireland [54].

Previous CG NBS cohort studies have discussed programme implementation, financial implications, technicalities, and acute outcomes, with some smaller studies addressing long‐term outcomes [55]. Our findings show a high survival rate (over 95%) for CG patients in Ireland and a significant increase in pre‐symptomatic diagnoses due to NBS. Despite NBSP success, symptomatic testing remains important for CG diagnosis, partly due to false negatives and potential metabolic decompensation before NBS results. Ongoing clinical education in early CG recognition and management is recommended to ensure regular up‐skilling of maternity and paediatric staff, even with NBSP.

### 4.1 Genotype

The prevalence of Q188R homozygosity in our cohort exceeds that previously reported in Caucasian populations [56, 57] but is lower than described in the Irish population by Coss et al. [17]. Possible reasons include increased ethnic diversity, declining ITC birth prevalence, and more frequent genetic testing in CG patients since the prior study ([58]). Q188R homozygosity was associated with cataracts in this study. These findings reinforce the evidence base that Q188R homozygosity is associated with a more severe clinical phenotype than other *GALT* variants.

### 4.2 Role of Early Screening

Acute symptoms at diagnosis (e.g., coagulopathy, transaminitis, sepsis), pointing towards a more severe form of CG and symptomatic therapy, were significantly associated with increased odds of childhood developmental issues. Diagnosis on HRS rather than NBS or symptomatic testing tended to improve outcomes, with lower odds of death and developmental issues; whether this finding is predominantly due to the lower incidence of acute intoxication symptoms, the use of lactose‐free feeds from birth, or the earlier age at diagnosis in this cohort remains unclear. Given the range of conditions screened for in the Republic of Ireland through the NBSP, earlier testing for CG would likely involve moving towards a biphasic NBSP, with early (Day 1–2) testing for intoxication‐type metabolic disorders and later (Day 5–10) testing for other conditions; however, based on our findings and practicability, changing the CG NBS timing cannot be recommended given the increase in screening burden this would incur, and insufficient data on whether the timing of HRS or feed type in high‐risk infants are the main drivers of improved outcomes. Instead, improving early detection may involve targeting HRS enrolment, which was 88% for eligible infants since 1996. Further qualitative research is needed to understand factors affecting enrolment, and further education of maternity staff, families with CG history, and ITC members is required to increase HRS enrolment. A pilot project for antenatal carrier testing in ITC women is underway in Ireland. If successful, this could reduce the number of infants needing HRS as well as parental anxiety and interference with breastfeeding, where possible.

### 4.3 Developmental Outcomes

Over the 50‐year study period, access to developmental screening, assessment, and early intervention has evolved. Learning and developmental issues in children with CG are common, with rates in our study (47.8%) aligning with previous reports. Inter‐study variability may be due to differing definitions, study periods, and screening standardisation. Comparison of these figures with LD and SLD prevalence in the general paediatric population is challenging due to variability in reported prevalence, definitions, and origin of report. A 2011 report by the National Council for Special education reported 25% of children having special educational needs of some form at age 7, while the Growing Up In Ireland study from 2018 reported 16.8% of children had a speech and language difficulty at 5 years [59, 60]. Comparison of LD, SLD, and motor issue prevalence in the ITC and non‐ITC populations showed a trend towards higher rates of developmental issues in the non‐ITC population. This is notable given the large body of evidence showing that the ITC population has a higher incidence of neurogenetic disorders, lower literacy, and higher deprivation rates than non‐ITC in the non‐CG general population [49, 50, 51].

Cerebellar, frontal grey matter, and diffuse white matter changes have been noted in CG, though the pathophysiology of developmental disorders is not well understood [61]. Regular, standardised developmental screenings should be offered per Galactosemia Network Guidelines [53]. Validated tools like the Ages & Stages‐3 questionnaire can help clinicians recognise atypical developmental trajectories and offer early intervention [62, 63]. The incidence of developmental issues has decreased over the course of the study period, with the year of birth associated with developmental issues. This may be related to increased understanding, evidence‐based care, and developmental interventions in later years, but also changes in cognitive assessment tools over the years.

The literature discusses motor planning pathways in CG, though their relevance to genetic diagnosis or complications is unclear. Possible causes may include cerebellar damage [37], transcallosal fibre alterations, or pallidum involvement [64]. Motor problems often co‐occur with speech and language impairments and other neurological issues in CG. Motor issue prevalence in children with CG ranges from 18%–52% [20, 27, 35, 36, 64, 65, 66]. In our cohort, distinct motor disorders (excluding FA) were found in 4.3% of patients, and in 11.9% when all motor disorders (including FA) were considered. The lower rate may be due to earlier years' screening limitations, as the relationship between CG and motor issues was not recognized. Most children with motor disorders also had speech and language delays and learning difficulties, supporting the hypothesis of a common cause.

Given specific speech, language, and motor skill deficits, comprehensive neuropsychological assessments should be considered at key times, such as school placement decisions and before state exams. Different cognitive functions develop at different times, so repeat assessments can inform neurodevelopmental trajectories and any widening skill gaps in CG. Children with FSIQ above 70 but below average may have deficits masked by standard tests like WISC 5th edition. In these cases, formal speech and language assessments are crucial to identify the nuanced expressive, receptive, grammatical, and word‐finding language deficits in CG. Executive functioning deficits may also be masked as emotional or behavioral issues and should be formally assessed using neuropsychological tools. CG patients often have lower white matter volume than controls, a finding common across neurodevelopmental disabilities, leading to deficits in cognitive performance, learning, and social skills [67]. In CG, dietary treatment from birth modifies white matter changes [68, 69]. Unidentified genetic and biochemical modifiers may contribute to this finding. Managing cognitive and executive function impacts should focus on scaffolding strengths, such as visual learning for those with language disruptions, and supplementing verbal learning when visuomotor skills are lacking [67].

### 4.4 Medical Comorbidities

The childhood prevalence of fractures in this study (3.2%) was significantly lower than reported figures of 30% for the general paediatric population [70]. The authors propose that this is due to insufficient documentation of previous fractures in this study rather than a lower true prevalence. Documentation and screening regarding bone health in our cohort were scarce in the early years of this study, likely due to limited evidence on this complication at the time. Intermittent DEXA scanning access over the 50‐year period is also a significant factor. Bone health assessment and osteopenia prevention evidence have evolved, recognising the role of Vitamin D, calcium supplementation, and bone density screening modalities, but DEXA access may still be limited.

The paucity of data on POI prohibits the drawing of any conclusions or recommendations on its screening, diagnosis, management, and prevention. POI was first described in the CG population in 1979 [71]. A paediatric gynaecology specialist service was made available to our cohort from 2008 for girls aged 12 and older. The true extent of POI in our cohort is unknown and would not be expected to be fully recorded in our centre's medical records. POI remains a known prevalent CG complication with significant lifelong medical and psychosocial consequences, and European Galactosaemia Network Guidelines should be followed for further management [53].

The prevalence of FA in our cohort exceeds that reported in the international CG population. These two separate autosomal recessive conditions (CG and FA), located on either side of chromosome nine's centromere, appear to be in linkage disequilibrium in this subgroup of affected individuals [72, 73, 74]. FA is a progressive neurological disorder characterised by ataxia, incoordination, sensory loss, and progressive neurological deterioration, accompanied by cardiac, endocrine, and orthopaedic sequelae. Symptoms typically onset in childhood, with an average age of 10 years. FA prevalence in Ireland has been estimated as the highest in Europe at 1 in 23 000 [75].

Cataracts were rare in this CG cohort, occurring in 8%. Cataract development was associated with later diagnosis, symptomatic testing, and Q188R homozygosity, highlighting the role of early diagnosis in preventing CG medical comorbidities. Ophthalmology follow‐up intervals in our cohort were suboptimal; engaging with patients and families to discuss follow‐up barriers and solutions is recommended. These barriers and solutions are likely specific to the area, culture, and health system, rather than CG‐related issues.

### 4.5 Strengths and Limitations

The strengths of this study are its large‐scale, population‐based, longitudinal design, describing the natural history of a classical cohort of patients. This provides the CG community with real‐life data from patients diagnosed and treated in a single national centre. The study timeline offers the research community insight into the impact of changes in investigation, complication recognition, and multidisciplinary management on CG patient outcomes in Ireland.

Data completeness is the main limitation, with 30.6% missing in primary outcomes; this is a recognised limitation of retrospective longitudinal studies [76]. The retrospective nature of our study means that investigation and management methodologies changed over time. Another limitation is the uneven application of standardised cognitive or neuropsychological assessments over the years.

## 5 Conclusion

This study represents the largest cohort of CG patients reported to date, providing a comprehensive analysis of the natural history, survival rates, and developmental outcomes of CG in a national paediatric cohort since NBSP was introduced. Over the time period studied here, NBS procedures and clinical care changed, as knowledge of CG progressed. Overall, a greater emphasis was placed on early diagnosis, HRS, NBSP follow‐up, early and comprehensive treatments, as well as the establishment of a dedicated galactosaemia clinic with a proactive multi‐disciplinary approach, in addition to needs‐based clinical interventions. The findings highlight the significant impact of early diagnosis and intervention on clinical outcomes, particularly through NBS and HRS. The high enrolment rates in the HRS program among ITC individuals emphasise the acceptability and importance of targeted screening efforts in high‐risk populations. The data show a high survival rate of over 95% among CG patients, with a notable reduction in mortality for those diagnosed on HRS. Despite these advances, the study reveals persistent challenges in achieving optimal long‐term outcomes. Developmental disorders, including learning difficulties, speech and language delays, and motor issues, affect nearly half of patients with CG; being diagnosed on HRS is protective. The association between acute CG symptoms at the time of diagnosis and developmental issues highlights the critical role of early detection and prompt dietary management in mitigating both acute and long‐term complications. Further efforts to embed developmental screening tools and early intervention are warranted.

To further improve outcomes, the study recommends ongoing clinical education for maternity and paediatric staff to ensure early recognition and management of symptomatic CG, even in the presence of an NBSP. Enhancing enrolment rates in the HRS program and exploring antenatal carrier testing in high‐risk populations could lead to earlier detection and better management of CG.

In conclusion, while the implementation of NBSP and HRS in Ireland has significantly improved survival rates and early diagnosis of CG, there remains a need for standardised developmental screening and early, multidisciplinary developmental intervention to address the high prevalence of developmental disorders. Continued efforts to refine screening programs and provide comprehensive care for CG patients will be essential in improving long‐term outcomes and quality of life for affected individuals, and the impact of innovative pre‐natal carrier testing on the need for HRS requires further study.

## Author Contributions

**D. Pereira:** study design, data collection, data analysis, manuscript writing, manuscript editing. **E. Loftus:** study design, data collection, manuscript editing. **C. E. Thompson:** study design, manuscript writing, manuscript editing. **F. Boyle:** manuscript writing, manuscript editing. **J. McNulty:** manuscript writing, manuscript editing. **R. Boruah:** manuscript editing. **E. Crushell:** data collection, manuscript editing. **C. Howard:** manuscript editing. **J. Hughes:** data collection, manuscript editing. **A. A. Monavari:** data collection, manuscript editing. **E. P. Treacy:** manuscript writing, manuscript editing. **A. Beegan:** statistical analysis, manuscript editing. **N. Jordan:** manuscript writing, manuscript editing. **Y. Rogers:** manuscript writing, manuscript editing. **A. Collins:** manuscript editing. **J. J. Brady:** data collection, manuscript editing. **M. Elsammak:** data collection, manuscript editing. **P. D. Mayne:** study design, data collection, manuscript editing. **I. Knerr:** study design, data collection, data analysis, manuscript writing, manuscript editing.

## Ethics Statement

Research and Ethics approval was obtained from the Childrens Health Ireland Research Ethics Committee for this study. Also, approval from the relevant national screening review boards was sought and granted.

## Consent

Individual patient consent statements were obtained as appropriate and where necessary, prior to publication.

## Conflicts of Interest

The authors declare no conflicts of interest.

## Data Availability

The data that support the findings of this study is available from the corresponding author upon reasonable request.

## References

[1] J. L. Fridovich‐Keil , “Galactosemia Reference Module in Life Sciences,” 2017, https://www.sciencedirect.com/science/article/abs/pii/B9780128096338064591.

[2] H. M. Holden , I. Rayment , and J. B. Thoden , “Structure and Function of Enzymes of the Leloir Pathway for Galactose Metabolism,” Journal of Biological Chemistry 278, no. 45 (2003): 43885–43888. 12923184 10.1074/jbc.R300025200

[3] M. Haskovic , A. I. Coelho , J. Bierau , et al., “Pathophysiology and Targets for Treatment in Hereditary Galactosemia: A Systematic Review of Animal and Cellular Models,” Journal of Inherited Metabolic Disease 43, no. 3 (2020): 392–408. 31808946 10.1002/jimd.12202 PMC7317974

[4] K. Coss , E. Treacy , E. Cotter , et al., “Systemic Gene Dysregulation in Classical Galactosaemia: Is There a Central Mechanism?,” Molecular Genetics and Metabolism 113, no. 3 (2014): 177–187. 25174965 10.1016/j.ymgme.2014.08.004

[5] A. Maratha , H. Colhoun , I. Knerr , K. Coss , P. Doran , and E. Treacy , “Classical Galactosaemia and CDG, the N‐Glycosylation Interface. A Review,” JIMD Reports 34, no. 1 (2017): 33–42. 27502837 10.1007/8904_2016_5 PMC5509556

[6] U. Mütze , K. Mengler , N. Boy , et al., “How Longitudinal Observational Studies Can Guide Screening Strategy for Rare Diseases,” Journal of Inherited Metabolic Disease 45, no. 5 (2022): 889–901. 35488475 10.1002/jimd.12508

[7] K. Schulpis , E. Papakonstantinou , H. Michelakakis , T. Podskarbi , A. Patsouras , and Y. Shin , “Screening for Galactosaemia in Greece,” Paediatric and Perinatal Epidemiology 11, no. 4 (1997): 436–440. 9373865 10.1046/j.1365-3016.1997.d01-31.x

[8] UKNSC , Screening for Galactosaemia: External Review Against Programme Appraisal Criteria for the UK National Screening Committee (UK NSC) (Bazian Ltd, Publishers, 2014), https://view‐health‐screening‐recommendations.service.gov.uk/galactosaemia/#:~:text=The%20UK%20NSC%20does%20not,misdiagnosed%20as%20having%20the%20condition.

[9] L. Varela‐Lema , L. Paz‐Valinas , G. Atienza‐Merino , R. Zubizarreta‐Alberdi , R. Villares , and M. López‐García , “Appropriateness of Newborn Screening for Classic Galactosaemia: A Systematic Review,” Journal of Inherited Metabolic Disease 39, no. 5 (2016): 633–649. 27116003 10.1007/s10545-016-9936-y

[10] N. Badawi , S. Cahalane , M. McDonald , et al., “Galactosaemia—A Controversial Disorder. Screening & Outcome. Ireland 1972‐1992,” Irish Medical Journal 89, no. 1 (1996): 16–17. 8984074

[11] A. Cohen , M. Baurek , A. Lund , M. Dunø , and D. Hougaard , “Including Classical Galactosaemia in the Expanded Newborn Screening Panel Using Tandem Mass Spectrometry for Galactose‐1‐Phosphate,” International Journal of Neonatal Screening 5, no. 2 (2019): 19. 33072978 10.3390/ijns5020019 PMC7510209

[12] M. Succoio , R. Sacchettini , A. Rossi , G. Parenti , and M. Ruoppolo , “Galactosemia: Biochemistry, Molecular Genetics, Newborn Screening, and Treatment,” Biomolecules 12, no. 7 (2022): 968. 35883524 10.3390/biom12070968 PMC9313126

[13] M. Pasquali , C. Yu , and B. Coffee , “Laboratory Diagnosis of Galactosemia: A Technical Standard and Guideline of the American College of Medical Genetics and Genomics (ACMG),” Genetics in Medicine 20, no. 1 (2018): 3–11. 29261178 10.1038/gim.2017.172

[14] R. Lak , B. Yazdizadeh , M. Davari , M. Nouhi , and R. Kelishadi , “Newborn Screening for Galactosaemia,” Cochrane Database of Systematic Reviews 12, no. 12 (2017): CD012272. 29274129 10.1002/14651858.CD012272.pub2 PMC6485983

[15] R. Lak , B. Yazdizadeh , M. Davari , M. Nouhi , and R. Kelishadi , “Newborn Screening for Galactosaemia,” Cochrane Database of Systematic Reviews 6, no. 6 (2020): CD012272. 32567677 10.1002/14651858.CD012272.pub3 PMC7387091

[16] L. Welling , A. Boelen , T. G. J. Derks , et al., “Nine Years of Newborn Screening for Classical Galactosemia in The Netherlands: Effectiveness of Screening Methods, and Identification of Patients With Previously Unreported Phenotypes,” Molecular Genetics and Metabolism 120, no. 3 (2017b): 223–228. 28065439 10.1016/j.ymgme.2016.12.012

[17] K. Coss , P. P. Doran , C. Owoeye , et al., “Classical Galactosaemia in Ireland: Incidence, Complications and Outcomes of Treatment,” Journal of Inherited Metabolic Disease 36, no. 1 (2013): 21–27. 22870861 10.1007/s10545-012-9507-9

[18] D. Demirbas , A. I. Coelho , M. E. Rubio‐Gozalbo , and G. T. Berry , “Hereditary Galactosemia,” Metabolism 83 (2018): 188–196. 29409891 10.1016/j.metabol.2018.01.025

[19] J. I. Malone , A. Diaz‐Thomas , and K. Swan , “Problems With the New Born Screen for Galactosaemia,” BMJ Case Reports (2011), 10.1136/bcr.01.2011.3769. PMC3109760 22693313

[20] M. Rubio‐Gozalbo , M. Haskovic , A. M. Bosch , et al., “The Natural History of Classic Galactosemia: Lessons From the GalNet Registry,” Orphanet Journal of Rare Diseases 14, no. 1 (2019): 86. 31029175 10.1186/s13023-019-1047-z PMC6486996

[21] I. Knerr , K. P. Coss , J. Kratzsch , et al., “Effects of Temporary Low‐Dose Galactose Supplements in Children Aged 5‐12 y With Classical Galactosemia: A Pilot Study,” Pediatric Research 78, no. 3 (2015): 272–279. 26053138 10.1038/pr.2015.107

[22] D. Milner , F. Boyle , J. McNulty , and I. Knerr , “Assessment of Dietary Intake of Iodine and Risk of Iodine Deficiency in Children With Classical Galactosaemia on Dietary Treatment,” Nutrients 15, no. 2 (2023): 407. 36678278 10.3390/nu15020407 PMC9860822

[23] P. J. Rutherford , D. C. Davidson , and S. M. Matthai , “Dietary Calcium in Galactosaemia,” Journal of Human Nutrition and Dietetics 15, no. 1 (2002): 39–42. 11903788 10.1046/j.1365-277x.2002.00330.x

[24] S. M. Thompson , F. E. Arrowsmith , and J. R. Allen , “Dietary Management of Galactosemia,” Southeast Asian Journal of Tropical Medicine and Public Health 34, no. Suppl 3 (2003): 212–214. 15906738

[25] B. van Erven , L. Welling , S. C. van Calcar , et al., “Bone Health in Classic Galactosemia: Systematic Review and Meta‐Analysis,” JIMD Reports 35, no. 1 (2017b): 87–96. 27995581 10.1007/8904_2016_28 PMC5585100

[26] L. A. Batey , C. K. Welt , F. Rohr , et al., “Skeletal Health in Adult Patients With Classic Galactosemia,” Osteoporosis International 24, no. 2 (2013): 501–509. 22525982 10.1007/s00198-012-1983-0

[27] S. E. Waisbren , N. L. Potter , C. M. Gordon , et al., “The Adult Galactosemic Phenotype,” Journal of Inherited Metabolic Disease 35, no. 2 (2012): 279–286. 21779791 10.1007/s10545-011-9372-y PMC3641771

[28] M. Thakur , G. Feldman , and E. E. Puscheck , “Primary Ovarian Insufficiency in Classic Galactosemia: Current Understanding and Future Research Opportunities,” Journal of Assisted Reproduction and Genetics 35, no. 1 (2018): 3–16. 28932969 10.1007/s10815-017-1039-7 PMC5758462

[29] R. A. Heidenreich , J. Mallee , S. Rogers , and S. Segal , “Developmental and Tissue‐Specific Modulation of Rat Galactose‐1‐Phosphate Uridyltransferase Steady State Messenger RNA and Specific Activity Levels,” Pediatric Research 34, no. 4 (1993): 416–419. 8255669 10.1203/00006450-199310000-00006

[30] M. E. Rubio‐Gozalbo , C. S. Gubbels , J. A. Bakker , P. P. C. A. Menheere , W. K. W. H. Wodzig , and J. A. Land , “Gonadal Function in Male and Female Patients With Classic Galactosemia,” Human Reproduction Update 16, no. 2 (2009): 177–188. 19793842 10.1093/humupd/dmp038

[31] J. L. Fridovich‐Keil , C. S. Gubbels , J. B. Spencer , R. D. Sanders , J. A. Land , and E. Rubio‐Gozalbo , “Ovarian Function in Girls and Women With GALT‐Deficiency Galactosemia,” Journal of Inherited Metabolic Disease 34, no. 2 (2011): 357–366. 20978943 10.1007/s10545-010-9221-4 PMC3063539

[32] B. van Erven , G. T. Berry , D. Cassiman , et al., “Fertility in Adult Women With Classic Galactosemia and Primary Ovarian Insufficiency,” Fertility and Sterility 108, no. 1 (2017a): 168–174. 28579413 10.1016/j.fertnstert.2017.05.013

[33] K. M. Antshel , I. O. Epstein , and S. E. Waisbren , “Cognitive Strengths and Weaknesses in Children and Adolescents Homozygous for the Galactosemia Q188R Mutation: A Descriptive Study,” Neuropsychology 18, no. 4 (2004): 658–664. 15506833 10.1037/0894-4105.18.4.658

[34] G. T. Berry and L. J. Elsas , “Introduction to the Maastricht Workshop: Lessons From the Past and New Directions in Galactosemia,” Journal of Inherited Metabolic Disease 34, no. 2 (2011): 249–255. 21116719 10.1007/s10545-010-9232-1

[35] S. Schweitzer , Y. Shin , C. Jakobs , and J. Brodehl , “Long‐Term Outcome in 134 Patients With Galactosaemia,” European Journal of Pediatrics 152, no. 1 (1993): 36–43. 8444204 10.1007/BF02072514

[36] D. Waggoner , N. Buist , and G. Donnell , “Long‐Term Prognosis in Galactosaemia: Results of a Survey of 350 Cases,” Journal of Inherited Metabolic Disease 13, no. 6 (1990): 802–818. 1706789 10.1007/BF01800204

[37] N. L. Potter , Y. Nievergelt , and L. D. Shriberg , “Motor and Speech Disorders in Classic Galactosemia,” JIMD Reports 11, no. 1 (2013): 31–41. 23546812 10.1007/8904_2013_219 PMC3755563

[38] L. J. Elsas and K. Lai , “The Molecular Biology of Galactosemia,” Genetics in Medicine 1, no. 1 (1998): 40–48. 11261429 10.1097/00125817-199811000-00009

[39] A. I. Coelho , M. E. Rubio‐Gozalbo , J. B. Vicente , and I. Rivera , “Sweet and Sour: An Update on Classic Galactosemia,” Journal of Inherited Metabolic Disease 40, no. 3 (2017): 325–342. 28281081 10.1007/s10545-017-0029-3 PMC5391384

[40] N. V. Guerrero , R. H. Singh , A. Manatunga , G. T. Berry , R. D. Steiner , and L. J. Elsas, II , “Risk Factors for Premature Ovarian Failure in Females With Galactosemia,” Journal of Pediatrics 137, no. 6 (2000): 833–841, 10.1067/mpd.2000.109148. 11113841

[41] F. R. Kaufman , J. K. V. Reichardt , W. G. Ng , et al., “Correlation of Cognitive, Neurologic, and Ovarian Outcome With the Q188R Mutation of the Galactose‐1‐Phosphate Uridyltransferase Gene,” Journal of Pediatrics 125, no. 2 (1994): 225–227. 8040766 10.1016/s0022-3476(94)70197-0

[42] K. Lai , S. D. Langley , R. H. Singh , P. P. Dembure , L. N. Hjelm , and L. J. Elsas , “A Prevalent Mutation for Galactosemia Among Black Americans,” Journal of Pediatrics 128, no. 1 (1996): 89–95. 8551426 10.1016/s0022-3476(96)70432-8

[43] E. Crushell , J. Chukwu , P. Mayne , J. Blatny , and E. Treacy , “Negative Screening Tests in Classical Galactosaemia Caused by S135L Homozygosity,” Journal of Inherited Metabolic Disease 32, no. 3 (2009): 412–415. 19418241 10.1007/s10545-009-1081-4

[44] Q. Katler , K. M. Stepien , N. Paull , et al., “A Multinational Study of Acute and Long‐Term Outcomes of Type 1 Galactosemia Patients Who Carry the S135L (c.404C>T) Variant of GALT,” Journal of Inherited Metabolic Disease 45, no. 6 (2022): 1106–1117. 36093991 10.1002/jimd.12556 PMC9643640

[45] Central Statistics Office , “*Live Births* 1972‐2022 Online: Central Statistics Office,” 2022, https://data.cso.ie/.

[46] Central Statistics Office , “*Census* 2016 Online: Central Statistics Office,” 2016, https://www.cso.ie/en/census/census2016reports/.

[47] Health Service Executive , HSE Intercultural Guide (2024), Dublin, 'Irish Travellers', https://www.hse.ie/eng/about/who/primarycare/socialinclusion/travellers‐and‐roma/irish‐travellers/.

[48] Department of Justice , “Traveller and Roma Policy in Ireland Online: Department of Justice,” 2022, http://www.travellerinclusion.ie/website/TravPolicy/travinclusionweb.nsf/page/anti_discrimination‐en.

[49] J. Barry and P. Kirke , “Congenital Anomalies in the Irish Traveller Community,” Irish Medical Journal 90, no. 6 (1997): 233–234. 9611926

[50] E. Forman , S. A. Lynch , I. Knerr , et al., “An Approach to Recognising and Identifying Metabolic Presentations in the Paediatric Irish Traveller Population,” European Journal of Pediatrics 182, no. 1 (2023): 31–40. 36374302 10.1007/s00431-022-04697-0

[51] Health Service Executive , National Traveller Health Action Plan 2022‐2027 (Department of Health, 2021), https://www.hse.ie/eng/services/publications/socialinclusion/national‐traveller‐health‐action‐plan‐2022‐2027.pdf.

[52] S. Neville , S. OSullivan , B. Sweeney , et al., “Friedreich Ataxia in Classical Galactosaemia,” JIMD Reports 26, no. 1 (2016): 1–5. 26219880 10.1007/8904_2015_477 PMC4864715

[53] L. Welling , L. E. Bernstein , G. T. Berry , et al., “International Clinical Guideline for the Management of Classical Galactosemia: Diagnosis, Treatment, and Follow‐Up,” Journal of Inherited Metabolic Disease 40, no. 2 (2017a): 171–176. 27858262 10.1007/s10545-016-9990-5 PMC5306419

[54] Gov.uk , Gypsy, Roma and Irish Traveller Ethnicity Summary (The National Archives, 2022), Online: Gov.uk, https://www.ethnicity‐facts‐figures.service.gov.uk/summaries/gypsy‐roma‐irish‐traveller#the‐gypsy‐roma‐traveller‐group.

[55] P. P. Jumbo‐Lucioni , “Diversity of Approaches to Classic Galactosemia Around the World: A Comparison of Diagnosis, Intervention, and Outcomes,” Journal of Inherited Metabolic Disease 35, no. 6 (2012): 1037–1049, 10.1007/s10545-012-9477-y. 22450714 PMC3774053

[56] J. L. Fridovich‐Keil and G. T. Berry , “Pathophysiology of Long‐Term Complications in Classic Galactosemia: What We Do and Do Not Know,” Molecular Genetics and Metabolism 137, no. 1 (2022): 33–39. 35882174 10.1016/j.ymgme.2022.07.005

[57] J. Lukac‐Bajalo , J. Marc , B. Mlinar , N. Karas , C. Krzisnik , and T. Battelino , “Frequencies of Q188R and N314D Mutations and IVS5‐24g>A Intron Variation in the Galactose‐1‐Phosphate Uridyl Transferase (GALT) Gene in the Slovenian Population,” Clinical Chemistry and Laboratory Medicine 40, no. 11 (2002): 1109–1113. 12521227 10.1515/CCLM.2002.194

[58] A. N. Hamid , J. Yurner , S. Abdalla , B. Quirke , L. Daly , and P. Fitzpatrick , “All‐Ireland Traveller Health Study,” 2010, https://www.gov.ie/en/publication/b9c48a‐all‐ireland‐traveller‐health‐study/.

[59] J. Banks and S. McCoy , A Study on the Prevalence of Special Educational Needs (NCSE and Economic & Social Institute, 2011), https://ncse.ie/wp‐content/uploads/2014/10/Prevalence_of_SEN_10_09_12.pdf.

[60] R. McConkey , A. Swift , and J. Titterington , “Changes in Children's Speech and Language Difficulties From Age Five to Nine: An Irish National, Longitudinal Study,” International Journal of Environmental Research and Public Health 18, no. 16 (2021): 8483. 34444228 10.3390/ijerph18168483 PMC8392088

[61] M. Hermans , M. Welsink‐Karssies , A. Bosch , K. Oostrom , and G. Geurtsen , “Cognitive Functioning in Patients With Classical Galactosemia: A Systematic Review,” Orphanet Journal of Rare Diseases 14, no. 1 (2019): 226. 31627760 10.1186/s13023-019-1215-1 PMC6798502

[62] S. Kendall , A. Nash , A. Braun , G. Bastug , E. Rougeaux , and H. Bedford , “Acceptability and Understanding of the Ages & Stages Questionnaires, Third Edition, as Part of the Healthy Child Programme 2‐Year Health and Development Review in England: Parent and Professional Perspectives,” Child: Care, Health and Development 45, no. 2 (2019): 251–256. 30661256 10.1111/cch.12639 PMC6849765

[63] K. Marks , S. N. Madsen , and P. Wilson , “Comparative Use of the Ages and Stages Questionnaires in the USA and Scandinavia: A Systematic Review,” Developmental Medicine and Child Neurology 61, no. 4 (2019): 419–430. 30246256 10.1111/dmcn.14044

[64] I. Rubio‐Agusti , M. Carecchio , K. P. Bhatia , et al., “Movement Disorders in Adult Patients With Classical Galactosemia,” Movement Disorders 28, no. 6 (2013): 804–810. 23400815 10.1002/mds.25348

[65] J. Hughes , S. Ryan , D. Lambert , et al., “Outcomes of Siblings With Classical Galactosemia,” Journal of Pediatrics 154, no. 5 (2009): 721–726. 19181333 10.1016/j.jpeds.2008.11.052

[66] F. Kaufman , C. McBride‐Chang , F. Manis , J. Wolff , and M. Nelson , “Cognitive Functioning, Neurologic Status and Brain Imaging in Classical Galactosemia,” European Journal of Pediatrics 154, no. 7 Suppl 2 (1995): s2–s5. 10.1007/BF02143794 7671958

[67] E. Cainelli , F. Arrigoni , and L. Vedovelli , “White Matter Injury and Neurodevelopmental Disabilities: A Cross‐Disease (Dis)connection,” Progress in Neurobiology 193 (2020): 101845. 32505757 10.1016/j.pneurobio.2020.101845

[68] B. Ahtam , S. E. Waisbren , V. Anastasoaie , et al., “Identification of Neuronal Structures and Pathways Corresponding to Clinical Functioning in Galactosemia,” Journal of Inherited Metabolic Disease 43, no. 6 (2020): 1205. 32592186 10.1002/jimd.12279

[69] M. Welsink‐Karssies , A. Schrantee , M. W. Caan , et al., “Gray and White Matter Are Both Affected in Classical Galactosemia: An Explorative Study on the Association Between Neuroimaging and Clinical Outcome,” Molecular Genetics and Metabolism 131, no. 4 (2020): 370–379. 33199205 10.1016/j.ymgme.2020.11.001

[70] C. Cooper , E. M. Dennison , H. G. Leufkens , N. Bishop , and T. P. van Staa , “Epidemiology of Childhood Fractures in Britain: A Study Using the General Practice Research Database,” Journal of Bone and Mineral Research 19, no. 1 (2004): 1976–1981. 15537440 10.1359/JBMR.040902

[71] D. Hoefnagel , D. Wurster‐Hill , and E. Child , “Ovarian Failure in Galactosaemia,” Lancet 2, no. 8153 (1979): 1197. 10.1016/s0140-6736(79)92430-9 91932

[72] L. Corben , V. Collins , S. Milne , et al., “Clinical Management Guidelines for Friedreich Ataxia: Best Practice in Rare Diseases,” Orphanet Journal of Rare Diseases 17, no. 1 (2022): 415. 36371255 10.1186/s13023-022-02568-3 PMC9652828

[73] M. J. Kelly , P. Bogdanova‐Mihaylova , J. Skeens , et al., “The Cost of Living With Inherited Ataxia in Ireland,” Cerebellum 21, no. 2 (2022): 280–296. 34228323 10.1007/s12311-021-01271-6 PMC8993771

[74] J. Schulz , S. Boesch , K. Bürk , et al., “Diagnosis and Treatment of Friedreich Ataxia: A European Perspective,” Nature Reviews. Neurology 5, no. 4 (2009): 222–234. 19347027 10.1038/nrneurol.2009.26

[75] P. Vankan , “Prevalence Gradients of Friedreich's Ataxia and R1b Haplotype in Europe Co‐Localize, Suggesting a Common Palaeolithic Origin in the Franco‐Cantabrian Ice Age Refuge,” Journal of Neurochemistry 126, no. Suppl 1 (2013): 11–20. 23859338 10.1111/jnc.12215

[76] C. Okpara , C. Edokwe , G. Ioannidis , A. Papaioannou , J. Adachi , and L. Thabane , “The Reporting and Handling of Missing Data in Longitudinal Studies of Older Adults Is Suboptimal: A Methodological Survey of Geriatric Journals,” BMC Medical Research Methodology 22, no. 1 (2022): 122. 35473665 10.1186/s12874-022-01605-w PMC9040343

